# Clusters in Behçet’s syndrome

**DOI:** 10.1186/s13075-022-02937-0

**Published:** 2022-10-29

**Authors:** Ufuk İlgen

**Affiliations:** Department of Rheumatology, Trakya University Medical School, 22030 Edirne, Turkey

**Keywords:** Behçet’s syndrome, Cluster analysis, Factor analysis, Phenotype

## Abstract

Clustering is an important clinical feature of Behçet’s syndrome (BS) and may have pathogenetic and therapeutic implications. Recent and previous studies on BS phenotype differ substantially in terms of methodology. Correlation matrices and factor analyses were not efficient enough to uncover clusters. Clustering patterns may change according to demographic factors such as age and sex. Clustering patterns may also be profoundly influenced by the misperception of symptoms that are assumed to be secondary to BS, when, in fact, they represent manifestations of BD mimics. This can give rise to misleading conclusions and should be kept in mind when interpreting data obtained by clustering or other phenotype analyses of BS. A true geographical/racial variability in disease expression could be studied in a multinational consensus cohort. Pathogenetic studies in separate clusters of BS have still been lacking.

**Supplementary Information:**

The online version contains supplementary material available at 10.1186/s13075-022-02937-0.

Recently, two studies from the Far East on clustering of clinical findings in Behçet’s syndrome (BS) were published in *Arthritis Research & Therapy* [1, 2]. Observation of clustering in clinically heterogeneous diseases is of importance and may have potential pathogenetic and therapeutic implications. Based on clinical findings, BS phenotypes such as skin-mucosa, joint, vascular, eye, neurological, and gastrointestinal involvement were previously defined with varying degrees of overlap from different parts of the world [3]. Although differing organ responses to different drugs suggested that pathogenetic background of BS phenotypes might differ [4], no differential pathogenetic mechanism was truly identified in separate BS phenotypes to date. The addition of *HLA-B* status, the most important genetic risk factor for BS, did not either ease clustering or simplify the clinical picture [5]. These may imply that clinical clustering is an overemphasized phenomenon in BS. Additionally, clustering methods themselves are prone to errors such as data processing and parameter selection that could potentially result in emergence of clusters which do not exist naturally [6]. Considering the rarity and heterogeneity of the disease and possibility of the mimics, particularly for certain phenotypes such as mucocutaneous-only and gastrointestinal disease, clustering patterns may be skewed to the extent of questionable reliability. However, clustering is an active area of BS research, and it should be noted that pathogenetic studies in separate clusters of BS (with reduced heterogeneity compared to the entire syndrome) have still been lacking preventing more conclusive interpretations. This commentary aims to explain discrepancies in clustering patterns in the two recent studies [1, 2] along with a methodological critique on previous works on BS phenotype.

Generated clusters in the two studies [1, 2] only partially overlapped although both were from the Far East. Notably, the solo skin-mucosa cluster, constituted by more than one third of all patients in the study from China [1], was not identified as a separate cluster in the study from Japan [2]. Almost half of the patients in the skin-mucosa-joint (cluster 1) and more than half in the skin-mucosa-eye (cluster 3) clusters in the latter but none in the skin-mucosa and joint clusters in the former study had eye involvement. Gastrointestinal cluster in the former did not include patients with eye, vascular, and joint involvement but one to two thirds of the patients in the gastrointestinal cluster (cluster 2) of the latter had eye, vascular, and joint disease. Neurological involvement was included in the cardiovascular cluster in the former but the neurological cluster (cluster 5) of the latter had almost no patients with vascular involvement. Besides some differences in the study populations such as those in uveitis and arthritis rates, the two studies differed methodologically as well. Although both used a statistical cluster analysis method, the Japanese study relied solely on the clinical manifestations while the Chinese study included the age, sex, disease duration, and severity in addition to the clinical manifestations in clustering. Since sex has a prominent effect on disease phenotype, its inclusion in clustering in the Japanese study may transfer patients with eye involvement in clusters 1 and 3 to the eye cluster (cluster 4) and remove patients with vascular and eye involvement from the gastrointestinal cluster (cluster 2). This may result in more similar pictures from the two studies. Not just for comparison purposes but for a proper understanding of clustering in BS and reproducibility, demographic features such as age and sex, which have been known to be closely associated with disease manifestations, should be included in cluster analyses. Geographical/racial variability in BS clustering may represent an artifact generated by flawed input into cluster analyses, and it is important to recognize that the resolution of this problem will not be brought about by improvement of the methodological analysis approach per se; its foundations may lie in the possible misperception of disease manifestations that may be due clinical entities that are not BS, therefore potentially biasing analyses. A multinational consensus cohort could be best to depict a true region/race-related clustering.

As discussed in the two articles [1, 2] and reviewed by Seyahi [3], several previous studies investigated the associations of clinical manifestations and identified phenotypes in BS. Most of these studies addressed the relationship between prespecified disease manifestations such as papulopustular lesions and arthritis, posterior uveitis and parenchymal neurological involvement, and uveitis and gastrointestinal involvement [3]. Only four attempted to take a panoramic picture of the whole syndrome with different methodological approaches [7–10]. Arida et al. [9] concluded that clusters did not exist in Greek patients with BS by applying pairwise correlations to nine clinical findings (oral ulcers, genital ulcers, erythema nodosum, folliculitis, arthritis, thrombophlebitis, ocular, gastrointestinal, and neurological involvement). Although presence of intercorrelations between clinical findings might ease clustering, it is not a prerequisite for cluster analysis and absence of such intercorrelations does not exclude clustering of the cases (Additional file 1). Factor analysis as a principal method was used in the other three [7, 8, 10]. Factor-based clustering was applied to its own extraction cohort in the study by Karaca et al. [10]. By such a strategy, 66.6% of patients in the cohort were included in clustering leaving one third out, although a total of only 2 (1.1%) patients were assigned to the deep vein thrombosis-superficial vein thrombosis cluster [10]. Uveitis and erythema nodosum-genital ulcer clusters, as suggested by the factor-based clustering idea, could either not be replicated in hierarchical cluster analysis [10].

Factor analysis was occasionally referred to as cluster analysis [3, 10, 11]. However, these two are conceptually different and not substitutes to each other. Factor analysis aims at simplification of complex data by transforming a set of variables to a set of factors, which are imaginary variables generated based on correlations of the original ones but reduced in number, still explaining a significant portion of the total variance [12]. Cluster analysis, on the other hand, is a way of meaningful categorization of the cases but not variables. In contrast to factor analysis, the number of clusters identified in a cluster analysis may exceed the number of variables since it is not a dimension reduction method (Additional file 1). Additionally, clustering may still be observed in datasets not appropriate for factor analysis. On a hypothetical BS cohort data (see Additional file 2 for the generation of the dataset in detail), it may be seen clearly how cluster and factor analyses do not translate to each other (Tables 1 and 2). Although patients with skin-mucosa involvement alone (or rarely with gastrointestinal involvement), the C1 cluster, constituted 30% of the entire cohort (Table 1), skin-mucosa involvement was a target for elimination in the factor analysis since it was relatively invariant (Table 2) (note that factor analysis is basically a variance analysis). If factor-based clusters were generated from and applied to the above-defined hypothetical BS cohort, more than half of all patients, constituting two large clusters, would be left out (Tables 1 and 2). While factor analysis is a useful way of determining associations of varying clinical findings, factor-based clustering is not an efficient way to uncover clusters. It also diverts attention away from relatively common findings. This makes comparison of recent [1, 2] and previous studies [7, 8, 10] quite difficult. As an example, absence of uveitis was identified as a separate factor (factor 3) in the study by Tunc et al. [8], and this imaginary variable was erroneously referred to as uveitis factor [8] and uveitis cluster [3]. However, it is apparently not possible to put BS patients without uveitis in a single clinical cluster.

**Table 1:** Characteristics and clustering of a hypothetical Behçet’s syndrome cohort

Involvement	C1	C2	C3	C4
Skin-mucosa	150 (100%)	145 (100%)	145 (100%)	45 (75%)
Joint	-	145 (100%)	5 (3.4%)	5 (8.3%)
Eye	-	-	145 (100%)	10 (16.7%)
Gastrointestinal	5 (3.3%)	-	-	50 (83.3%)
Vascular	-	-	-	55 (91.7%)
Neurological	-	-	-	55 (91.7%)
**Total**	150	145	145	60

Data were expressed as numbers (% within clusters)

See Additional file 2 for the generation of the cohort in detail

**Table 2:** Factors extracted from the rotated component matrices

	**Skin-mucosa included**			**Skin-mucosa eliminated**		
**Factor eigenvalues**	2.971	1.412		2.884	1.402	
**Variance explained (%)**	49.5	23.5		57.7	28	
**Loadings**	**Factor 1**	**Factor 2**	**Com**	**Factor 1**	**Factor 2**	**Com**
Skin-mucosa	− 0.346	0.193	0.157	-	-	-
Joint	− 0.241	**0.784**	0.673	− 0.247	− **0.826**	0.743
Eye	− 0.173	− **0.872**	0.790	− 0.204	**0.848**	0.761
Gastrointestinal	**0.948**	0.027	0.899	**0.966**	0.016	0.933
Vascular	**0.965**	0.042	0.933	**0.961**	− 0.027	0.924
Neurological	**0.964**	− 0.046	0.931	**0.959**	0.062	0.924

Kaiser–Meyer–Olkin Measure of Sampling Adequacy = 0.688 (skin-mucosa included) and 0.742 (skin-mucosa eliminated), *p* < 0.001 for the Bartlett’s Tests of Sphericity for both. Com, communalities

See Additional file 2 for the generation of the cohort in detail

Lastly, BS manifestations active in the last three months in the studies by Tunc [8] and Karaca et al. [10] but cumulative presence of manifestations in the two current [1, 2] and previous studies [7, 9] were taken into account in the assessments. Considering natural disease course, observation period, and impact of treatment on disease phenotype, this issue may also be a source of discrepancy.

In conclusion, clustering is an important clinical feature of BS. Recent and previous studies on BS phenotype differ substantially in terms of methodology preventing proper comparisons. Clustering pattern may change according to demographic factors such as age and sex and factors that include possible misclassification of disease manifestations as BS-compatible or the constellation of BD manifestations that are more likely to represent a different condition such as Stevens-Johnson syndrome-like eruptions or inflammatory bowel disease. Geographical/racial variability in disease expression could be studied in a multinational consensus cohort. Pathogenetic studies in separate clusters of BS have still been lacking.

Methodological Note: IBM SPSS Statistics for Windows v.21.0 (IBM Corp., Armonk, NY, USA) was used for the statistical analyses. The same cluster and factor analysis methods reported by Zou [1] and Karaca et al. [10] were applied in this commentary.

## Supplementary Information

**Additional file 1.**

**Additional file 2.**

## Data Availability

The derived data generated in this research will be shared on reasonable request to the corresponding author.

## Abbreviation

BS: Behçet’s syndrome
