# Supplementary material for: Clusters in Behçet’s syndrome
Source: Arthritis Res Ther. 2022 Oct 29;24:242. doi: 10.1186/s13075-022-02937-0 (PMC9617315; doi:10.1186/s13075-022-02937-0)
Supplement: Supplementary file 1 — Additional file 1. [file 13075_2022_2937_MOESM1_ESM.pdf]

Pairwise correlations and clustering

Let A, B, and C be the three clinical findings of a disease and assume that no pairwise correlations exist. If 8*n* cases were distributed according to the presence or absence of the clinical findings as represented in Panel A, the number of cases in each possible status would be the same and equal to *n*. Since all eight possible disease phenotypes had the same frequency, i.e. the cases clustered equally in all possible situations, there would be no need for further analysis in terms of clustering. But if the cases were distributed as represented in Panel B without violating the assumption of absence of any pairwise correlations, four phenotypic clusters (C1-4) would be identifiable with equal frequencies. In conclusion, absence of intercorrelations of the clinical findings does not exclude clustering of the cases.

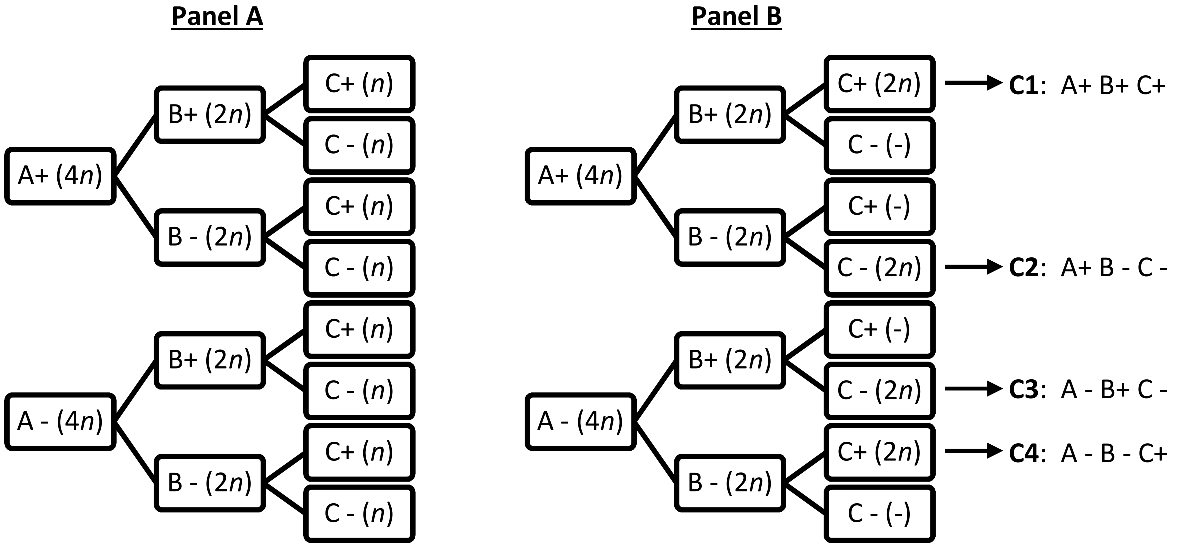

Figure i. Two possible distributions of 8*n* cases according to the presence or absence of the clinical findings (A, B, and C) without violating the assumption of absence of any pairwise intercorrelations.
