# Supplementary material for: Clusters in Behçet’s syndrome
Source: Arthritis Res Ther. 2022 Oct 29;24:242. doi: 10.1186/s13075-022-02937-0 (PMC9617315; doi:10.1186/s13075-022-02937-0)
Supplement: Supplementary file 2 — Additional file 2. [file 13075_2022_2937_MOESM2_ESM.pdf]

Generation of a hypothetical Behçet's syndrome cohort

It was intended to create a hypothetical dataset of Behçet's syndrome (BS) patients (Dataset1) with pre-defined characteristics of 1) skin-mucosa 2) joint 3) eye 4) gastrointestinal 5) vascular and 6) neurological involvement aiming at an extreme clustering and association under four phenotypes: skin-mucosa alone (C1), skin-mucosa and joint (C2), skin-mucosa and eye (C3), and skin-mucosa and internal organ (gastrointestinal, vascular, and neurological) involvement (C4). The data were represented in Table i.

Table i. Characteristics and clustering of a hypothetical Behçet's syndrome cohort (derived from Dataset1).

Involvement	C1	C2	C3	C4
Skin-mucosa	150 (100%)	150 (100%)	150 (100%)	50 (100%)
Joint	-	150 (100%)	-	-
Eye	-	-	150 (100%)	-
Gastrointestinal	-	-	-	50 (100%)
Vascular	-	-	-	50 (100%)
Neurological	-	-	-	50 (100%)
**Total**	150	150	150	50

Data were expressed as numbers (% within clusters).

As is evident from the table, clustering of the cases and association between the variables are simple and clear, and do not require any further analysis. Then, the dataset was distorted towards a more real one (Dataset2) by addition of some minor associations without a salient impact on the clusters (Table ii).

Table ii. Characteristics of the clusters after addition of some minor associations (derived from Dataset2).

Involvement	C1	C2	C3	C4
Skin-mucosa	150 (100%)	145 (96.7%)	145 (96.7%)	45 (90%)
Joint	-	150 (100%)	5 (3.3%)	-
Eye	-	-	150 (100%)	5 (10%)
Gastrointestinal	5 (3.3%)	-	-	50 (100%)
Vascular	-	5 (3.3%)	-	50 (100%)
Neurological	-	-	5 (3.3%)	50 (100%)
**Total**	150	150	150	50

Data were expressed as numbers (% within clusters).

If a confirmatory analysis is run by the TwoStep Cluster, a thiny fifth cluster may even be identifiable in the new dataset (Figure i). However, since the fifth cluster was quite small and heterogeneous, the TwoStep Cluster analysis was modified by limiting the number of clusters to four, and resulted in a clustering pattern similar to the initial one (Table iii, the final table).

Figure i. Analysis of Dataset2 by TwoStep Cluster and identification of a new cluster (C5).

**Involvement**	**C1**	**C2**	**C3**	**C4**
Skin-mucosa	150 (100%)	145 (96.7%)	145 (96.7%)	45 (90%)
Joint	-	150 (100%)	5 (3.3%)	-
Eye	-	-	150 (100%)	5 (10%)
Gastrointestinal	5 (3.3%)	-	-	50 (100%)
Vascular	-	5 (3.3%)	-	50 (100%)
Neurological	-	-	5 (3.3%)	50 (100%)
**Total**	150	150	150	50

C5: 5 5 5 5

**Involvement**	**C1**	**C2**	**C3**	**C4**	**C5**
Skin-mucosa	150 (100%)	145 (100%)	140 (100%)	45 (100%)	5 (25%)
Joint	-	145 (100%)	-	-	10 (50%)
Eye	-	-	140 (100%)	-	15 (75%)
Gastrointestinal	5 (3.3%)	-	-	45 (100%)	5 (25%)
Vascular	-	-	-	45 (100%)	10 (50%)
Neurological	-	-	-	45 (100%)	10 (50%)
**Total**	150	145	140	45	20

Data were expressed as numbers (% within clusters). Dashed arrows indicate shared cases.

Table iii. Characteristics and clustering of the BS cohort after limiting the number of clusters to four (derived from Dataset2).

**Involvement**	**C1**	**C2**	**C3**	**C4**
Skin-mucosa	150 (100%)	145 (100%)	145 (100%)	45 (75%)
Joint	-	145 (100%)	5 (3.4%)	5 (8.3%)
Eye	-	-	145 (100%)	10 (16.7%)
Gastrointestinal	5 (3.3%)	-	-	50 (83.3%)
Vascular	-	-	-	55 (91.7%)
Neurological	-	-	-	55 (91.7%)
**Total**	150	145	145	60

Data were expressed as numbers (% within clusters).
